# Reducing urban-rural disparities in maternal and child mortality in China: a 33-year analysis and projection to 2030

**DOI:** 10.7189/jogh.16.04011

**Published:** 2026-01-12

**Authors:** Zuyao Rao, Bei Liu, Dehai Li, Zemin Qing, Yuxuan Lu, Dandan Yin, Shen Li, Kai Cheng, Yunpengcheng Xiao, Qiong Dai

**Affiliations:** 1 Department of Health, Maternal and Child Health Hospital of Hubei Province, Tongji Medical College, Huazhong University of Science and Technology, Wuhan, China; 2 Department of health education, Jinan Health Publicity and Education Center, Jinan, China

## Abstract

**Background:**

Despite substantial progress in maternal and child health (MCH) in China, achieving equitable outcomes across urban and rural areas remains elusive. To this end, this study investigated long-term trends in urban-rural disparities in maternal and child mortality rates in China from 1991 to 2023 and projected future trajectories for the 2024–30 period.

**Methods:**

We obtained data on national, urban, and rural maternal mortality ratios (MMRs), under-five mortality rates (U5MRs), infant mortality rates (IMRs), and neonatal mortality rates (NMRs) for the 1991–2023 period from the National Bureau of Statistics of China. We analysed and predicted mortality rates during this period by utilising rate differences (RDs), rate ratios (RRs), average growth rates, and estimated annual percentage changes, along with the autoregressive integrated moving average model.

**Results:**

From 1991 to 2023, the national MMRs declined from 80 per 100 000 live births to 15.1 per 100 000 live births. Over the same period, U5MRs decreased from 61.0‰ to 6.2‰, IMRs declined from 50.2‰ to 4.5‰, and NMRs declined from 33.1‰ to 2.8‰. During this period, urban MMRs decreased from 46.3 per 100 000 live births to 12.5 per 100 000 live births. Correspondingly, urban U5MRs declined from 20.9‰ to 3.9‰, IMRs declined from 17.3‰ to 2.9‰, and NMRs declined from 12.5‰ to 1.7‰. In rural areas, MMRs declined from 100 per 100 000 live births to 17.0 per 100 000 live births, while U5MRs declined from 71.1‰ to 7.2‰, IMRs declined from 58.0‰ to 5.2‰, and NMRs declined from 37.9‰ to 3.2‰. The RDs and RRs of MMRs, U5MRs, IMRs, and NMRs exhibited overall downward trends, while the autoregressive integrated moving average model predicted continued declines in mortality rates across the country, including in urban and rural areas, from 2024 to 2030.

**Conclusions:**

China has achieved substantial progress in MCH, with mortality rates and disparities falling in both urban and rural areas, yet urban-rural disparities persist. Future MCH services should shift from broad coverage to precision quality improvement. These experiences also offer valuable insights for low- and middle-income countries (LMICs) undergoing rapid urbanisation, highlighting the importance of coordinated development of urban and rural health systems to achieve equitable and accessible health outcomes.

Maternal and child health (MCH) is a cornerstone of global public health, directly linked to broader development goals such as poverty reduction, education, and gender equality [1]. Key MCH indicators, such as the maternal mortality ratio (MMR), under-five mortality rate (U5MR), infant mortality rate (IMR), and neonatal mortality rate (NMR), reflect both the quality of healthcare systems and the effectiveness of social welfare mechanisms [1,2]. Globally, achieving the United Nations Sustainable Development Goal (SDG) 3, which aims to reduce MMR to below 70 per 100 000 live births, the U5MR to below 25 per 1000 live births, and the NMR to below 12 per 1000 live births, remains a significant challenge [3]. As of 2023, data from low- and middle-income countries (LMICs) show MMRs ranging from 200 to 300 per 100 000 live births and IMRs between 20 and 40 per 1000 live births [4]. In LMICs, such as India and Brazil, systemic barriers, including inadequate infrastructure, unequal resource distribution, and social determinants of health, contribute to high maternal and child mortality rates [5]. India has made notable strides through initiatives such as the National Health Mission [6]; however, rural areas within the country have nearly 3-fold higher MMRs compared to urban ones, as well as substantially higher U5MRs and NMRs. Similarly, while Brazil’s national health initiatives have improved overall conditions (*e.g.* economic, education, and health policy), its rural areas, particularly the Amazon region, still experience twice higher MMRs than urban centres [7]. These persistent discrepancies underscore enduring global inequalities in MCH, necessitating coordinated international efforts and showing that even targeted interventions do not necessarily contribute to achieving universal health coverage and equitable outcomes.

China has made substantial progress in reducing MMRs, U5MRs, IMRs, and NMRs, particularly following late 20th-century economic growth and health reforms such as the New Rural Cooperative Medical Scheme, aligning with SDG 3 [8]. According to the National Health Commission, MMRs dropped from approximately 1500 to 12.5 per 100 000 live births, U5MRs decreased from 40 to 6.2 per 1000 live births, IMRs decreased from around 200 to 2.8 per 1000 live births, and NMRs fell from 50–70 to 1.7 per 1000 live births between pre-1949 and 2023. These accomplishments demonstrate the efficacy of targeted health policies and investments in maternal-child health services and highlight the importance of government commitment and resource mobilisation in achieving sustainable health improvements.

However, significant challenges persist in bridging China’s urban-rural health gap [9]. As of 2023, rural areas exhibit stark disparities: MMR is approximately 35% higher, U5MR is nearly twice higher, IMR is 79% higher, and NMR is 1.9 times higher than in urban areas. These ongoing urban-rural gaps emphasise the need for integrated approaches that align national policy with adaptive local implementation, positioning equitable healthcare access as a core policy objective. To further advance MCH, China has implemented long-term policies such as the Healthy China 2030 Plan [10], the China National Program for Women’s Development (2021–30), and the China National Program for Child Development (2021–30) [11], aiming to address disparities and promote social equity through enhanced healthcare delivery in underserved areas.

While numerous studies have analysed trends in maternal and child mortality, often relying on national averages that obscure subnational variation or neglecting to incorporate policy contexts, few have comprehensively examined urban-rural disparities in MCH over extended periods or projected future trajectories [12]. Here, we addressed existing gaps by analysing 1991–2023 trends in China’s urban-rural maternal and child mortality, utilising rate differences (RDs), rate ratios (RRs), average growth rates (AGRs), and estimated annual percentage changes (EAPCs) to quantify disparities and dynamically track the evolution of urban-rural gaps. Unlike previous short-term analyses [13], we used continuous long-term time series data to capture policy’s lag effects and cumulative impacts. For trend projection, we utilised an autoregressive integrated moving average (ARIMA) model to address data volatility and ensure residual conformity to white noise, thus improving forecast accuracy and overcoming traditional linear regression’s global linearity assumption [14].

## METHODS

We obtained the data on national, urban and rural MMRs, U5MRs, IMRs, and NMRs from the National Bureau of Statistics (NBS) of China from 1991 to 2023 [15]. The MMR refers to the number of maternal deaths per 100 000 live births in a given year. The NMR, IMR, and U5MR are defined as the number of deaths occurring before 28 days, 1 year, and 5 years of age, respectively, per 1000 live births in a given year.

The maternal and child health data within this national dataset were collected through China’s National Maternal and Child Mortality Surveillance System, which employed stratified cluster random sampling to select monitoring units, along with stringent reporting and quality control measures to ensure data accuracy and integrity. The final data set encompassed 31 provinces and municipalities across China, representing both urban and rural areas.

In China, urban areas consist of prefecture-level municipalities and municipalities directly under the jurisdiction of the central government, and rural areas comprise counties and county-level cities. Data on township health centres and village health offices are categorised within the statistics for the rural region.

The data on the annual MMR, U5MR, IMR, and NMR in China from 1991 to 2023 at the national level and separately in urban and rural areas are publicly available without personal identifying information; therefore, we did not require separate ethics approval or informed consent for our analysis.

### RD, RR, AGR, and EAPC

We assessed the inequality between urban and rural mortality using RDs and RRs, calculated as follows [16]:

RD = *R*_Rural_ – *R_U_*_rban_

RR = *R*_Rural_/*R_U_*_rban_,

Here, *R*_Rural_ and *R_U_*_rban_ represent mortality rates (MMR, U5MR, IMR, or NMR) in rural and urban areas, respectively.

We employed the geometric method to calculate AGR using the following formula:



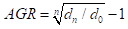



Here, *d*_0_ represents the base period index and *d_n_* represents the NTH year index. The disparities in AGR between urban and rural areas were indicated by GAP-1 and GAP-2, with their respective formula as follows: GAP-1 = (AGR_Urban_)/(AGR_Rural_), GAP-2 = ((AGR_Urban_) − (AGR_Rural_))/(AGR_Rural_).

The EAPC serves as a widely utilised summary measure for quantifying trends over a specified interval [17]. We fitted a linear regression model to the natural logarithm of the four MCH indicators (MMR, U5MR, IMR, and NMR). Specifically, the model could be expressed as: y = b + ax, where y = ln (MMR, U5MR, IMR, or NMR) and x = calendar year. We calculated the EAPC as: EAPC = 100 × (e^a^ − 1), and its 95% confidence interval (CI) could be obtained from a linear regression model.

### Autoregressive integrated moving average model

The ARIMA model, used for time series analysis and prediction, treats the data series of predicted objects over time as a random sequence [18]. It can be classified into three types: simple ARIMA (p, d, q) model, seasonal ARIMA (P, D, Q) S model, and seasonal-product ARIMA (p, d, q) (P, D, Q) S model, where p, d, q and P, D, Q are the orders of the non-seasonal and seasonal autoregressive terms, differencing terms, and moving average terms, respectively. This model can extract nonstationary deterministic information from a time series by calculating differences. When the residual sequence of an ARIMA model is random (white noise), the model is considered the best linear prediction model for short-term predictions of a time series. The ‘auto.arima()’ function in *R* is used to automatically select the optimal set of parameters for model construction by comparing the combinatorial spectrum of parameters based on the minimum Akaike’s information criterion (AIC) or Bayesian information criterion (BIC). While ‘auto.arima()’ returns only the optimal final model, the selection process itself is deterministic and reproducible. We used BIC in model selection, and the Ljung–Box test to assess whether the residual sequence can be considered a white noise sequence (*P* > 0.05 if the residual sequence is a white noise sequence). To validate the predictive performance of the ARIMA model, we compared the predicted mortality rates for 2023 with the actual observed values. Using annual mortality rate data from 1991 to 2023, we generated forecasts for the period 2024–30 using the ARIMA model. To ensure biological plausibility, we constrained the lower bound of the predicted mortality rate and its 95% CI to be no less than 20% of the historical data range in this study.

To address very low, but nonzero mortality rates, we applied a logarithmic transformation with an epsilon adjustment (ε = 0.01) to mitigate negative values [19]. To rigorously assess the robustness of ε = 0.01, we performed a sensitivity analysis as follows: we chronologically divided the dataset from 1991 to 2023 into a training set (1991–2018) and a validation set (2019–23). For each health indicator, we systematically evaluated three epsilon values (ε∈ {0.01, 0.1, 1}). For each ε, we transformed the training data *y*′ = ln (*y* + ϵ), followed by ARIMA model fitting with BIC-optimised parameters. Furthermore, we generated, inverted and truncated forecasts for 2019–23, and quantified model performance using mean absolute error (MAE) and root mean squared error (RMSE), enabling a comparative evaluation of robustness across ε values [20]. The optimal ε value should be selected based on minimising both MAE and RMSE metrics. When multiple ε values demonstrated comparable performance (defined as error differences <5%), we prioritised ε = 0.01 to prevent over-smoothing artefacts. To statistically verify the error distributions across ε values, the Friedman test was recommended, using a *P*-value threshold of 0.05 to reject the null hypothesis of equivalence.

### Statistical analysis

We used *R*, version 4.4.3 (R Core Team, Vienna, Austria) for the relevant statistical analysis. We considered a *P*-value less than 0.05 to be statistically significant in all analyses.

## RESULTS

### Descriptive analysis of MMR, U5MR, IMR, and NMR

**Figure 1:**
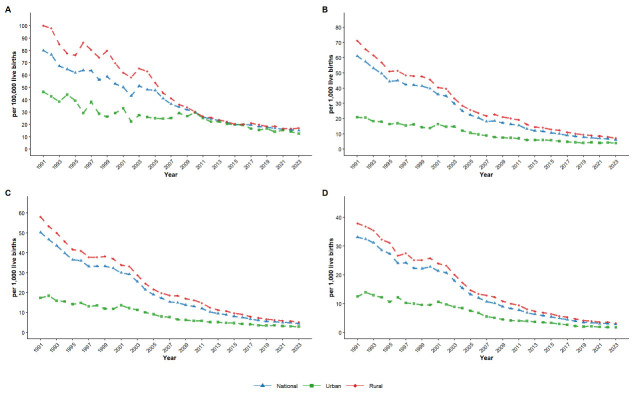
Change trends of health indicators at national, urban and rural levels. **Panel A.** Change the trends of MMR. **Panel B.** Change trends of U5MR. **Panel C.** Change trends of IMR. **Panel D**. Change trends of NMR.

The MMR, U5MR, IMR, and NMR in China showed overall downward trends at the national level and in urban and rural areas from 1991 to 2023 (**Figure 1**). The MMRs per 100 000 live births in national, urban and rural areas decreased from 80, 46.3, and 100 in 1991 to 15.1, 12.5, and 17.0 in 2023, respectively. The U5MRs of national, urban and rural areas decreased from 61.0‰, 20.9‰ and 71.1‰ in 1991 to 6.2‰, 3.9‰ and 7.2‰ in 2023, respectively. The IMRs of national, urban and rural areas decreased from 50.2‰, 17.3‰ and 58.0‰ in 1991 to 4.5‰, 2.9‰ and 5.2‰ in 2023, respectively. The NMRs of national, urban and rural areas decreased from 33.1‰, 12.5‰ and 37.9‰ in 1991 to 2.8‰, 1.7‰ and 3.2‰ in 2023, respectively (Table S1 in the **Online Supplementary Document**).

### RD and RR of MMR, U5MR, IMR, and NMR

**Figure 2:**
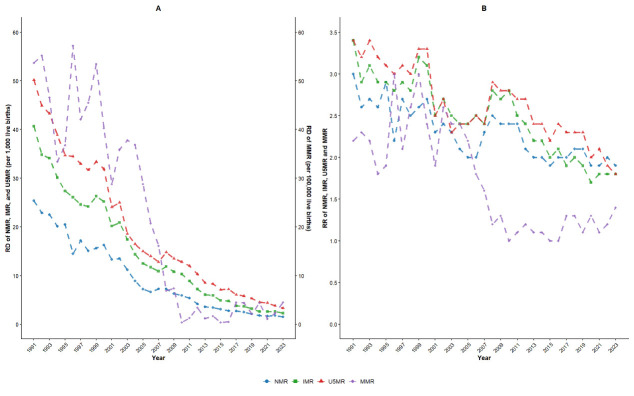
Trends of RD and RR of health indicators in China from 1991 to 2023. **Panel A.** Trends of RD of MMR, U5MR, IMR, and NMR. **Panel B**. Trends of RR of MMR, U5MR, IMR, and NMR.

The RDs and RRs of U5MR, IMR and NMR exhibited overall downward trends during the study period. However, the RDs of MMRs per 100 000 live births gradually decreased from 53.7 in 1991 to 0.4 in 2010, followed by fluctuations between 2011 and 2023 (ranging from 0.4 to 4.5). Meanwhile, RRs of MMRs decreased from 2.2 in 1991 to 1.0 in 2010 and then remained relatively stable between 2011 and 2023 (ranging from 1.0 to 1.4) (**Figure 2** and Table S2 in the **Online Supplementary Document**).

### AGR of MMR, U5MR, IMR, and NMR

From 1991 to 2023, the AGRs of national, urban, and rural MMRs were −5.08%, −4.01%, and −5.39%, respectively. Similarly, the AGRs of national, urban and rural U5MRs were −6.90%, −5.11%, and −6.91%, respectively; those of national, urban and rural IMRs were −7.26%, −5.43%, and −7.26%, respectively; and those of national, urban and rural NMRs were −7.43%, −6.04%, and −7.43%, respectively. The results of GAP analysis revealed that the decline rates in cities were slower compared to rural areas, with statistically significant differences (*P* < 0.05) (**Table 1**).

**Table 1:** The urban-rural disparities in the AGR of health indicators in China from 1991 to 2023*

Indicators	Urban AGR, %	Rural AGR, %	GAP-1 (95% CI)	GAP-2, % (95% CI)	Z_GAP-1_	Z_GAP-2_	*P*_GAP-1_	*P*_GAP-2_
**MMR**	−4.01	−5.39	0.74 (0.59, 0.92)	−25.60 (−32.60, −18.60)	−2.56	−2.49	0.010	0.013
**U5MR**	−5.11	−6.91	0.74 (0.59, 0.92)	−26.05 (−33.05, −19.05)	−2.61	−2.48	0.009	0.013
**IMR**	−5.43	−7.26	0.75 (0.60, 0.93)	−25.21 (−32.21, −18.21)	−2.52	−2.47	0.012	0.013
**NMR**	−6.04	−7.43	0.81 (0.67, 0.99)	−18.71 (−28.71, −8.71)	−2.11	−1.99	0.035	0.035

CI – confidence interval, IMR – infant mortality rate, MMR – maternal mortality ratio, NMR – neonatal mortality rate, U5MR-under-five mortality rate

*GAP-1 reflects the multiple relationships of urban and rural growth rates, making it appropriate for long-term trend analysis. GAP-2 highlights the difference in improvement speed between urban and rural areas, rendering it suitable for evaluating policy effectiveness.

### EAPC of MMR, U5MR, IMR, and NMR

From 1991 to 2023, the EAPCs of national, urban and rural MMRs were −5.35% (−5.64%, −5.07%), −3.34% (−3.73%, −2.86%), and −6.20% (−6.57%, −5.73%), respectively. Similarly, the EAPCs of national, urban and rural U5MRs were −7.23% (−7.50%, −6.95%), −5.64% (−5.92%, −5.26%), −7.04% (−7.23%, −6.76%), respectively; those of national, urban and rural IMRs were −7.60% (−7.97%, −7.32%), −5.82% (−6.11%, −5.54%), −7.50% (−7.78%, −7.23%), respectively; and those of national, urban and rural NMRs were −7.97% (−8.33%, −7.69%), −6.76% (−7.23%, −6.29%), −7.87% (−8.15%, −7.50%), respectively.

### Best-fitted selected ARIMA models for forecasting mortality rate

This research discovered that some distinct ARIMA models at national, urban, and rural levels had the best data fit by comparing AIC, BIC, and Ljung-Box Q and ensuring that the *P*-value is more than 0.05 if the residual sequence is a white noise sequence, as recommended in the literature. For instance, the optimal models selected were ARIMA (0, 1, 0) for national and rural MMR trends, while ARIMA (5, 2, 0) provided the best fit for national and rural U5MR and IMR. All selected models passed the white noise diagnostic, validating their adequacy for forecasting (**Table 2**). The results of residual analysis for MMR, U5MR, IMR, and NMR at the national, urban, and rural levels revealed randomly distributed model residuals with no discernible trends or patterns. Plots of autocorrelation (ACF) and partial autocorrelation (PACF) functions showed no significant serial correlation, while quantile-quantile (Q-Q) plots indicated approximate normality of the residuals (Figures S1–4 in the **Online Supplementary Document**).

**Table 2:** The best-fitted ARIMA models for forecasting the mortality rate

Indicators	Region	ARIMA (p, d, q)	AIC	BIC	R^2^	Ljung-Box Q	*P-*value	White noise test
**MMR**	National	(0, 1, 0)	178.67	181.61	−0.05	17.88	5.71	Passed
	Urban	(2, 1, 0)	174.34	180.21	0.46	7.96	6.33	Passed
	Rural	(0, 1, 0)	194.04	196.97	0.03	15.09	1.29	Passed
**U5MR**	National	(5, 2, 0)	106.75	115.35	0.61	6.96	7.29	Passed
	Urban	(1, 1, 0)	91.86	96.25	0.12	5.18	8.79	Passed
	Rural	(5, 2, 0)	111.41	120.02	0.70	5.94	8.21	Passed
**IMR**	National	(5, 2, 0)	90.32	98.93	0.62	8.19	6.09	Passed
	Urban	(1, 1, 0)	79.09	83.49	0.18	9.97	4.43	Passed
	Rural	(5, 2, 0)	104.04	112.64	0.58	9.24	5.09	Passed
**NMR**	National	(4, 2, 1)	74.49	83.09	0.56	5.30	8.70	Passed
	Urban	(1, 1, 0)	71.05	75.45	0.14	10.87	3.68	Passed
	Rural	(0, 2, 3)	94.07	99.81	0.37	8.35	5.94	Passed

AIC – Akaike’s information criterion, ARIMA – autoregressive integrated moving average, BIC – Bayesian information criterion, IMR – infant mortality rate, MMR – maternal mortality ratio, NMR – neonatal mortality rate, U5MR – under-five mortality rate

### Prediction of maternal and child mortality rates based on the best-fitted ARIMA model

We observed that the actual maternal and child mortality rates in 2023 closely align with these values, with all actual values falling within the 95% confidence interval (CI) of the predictions (Table S3 in the **Online Supplementary Document**).

We summarised the prediction errors of each health indicator under different ϵ values. We found that ϵ = 0.01 performed the best across most indicators, thereby demonstrating its robustness (Table S4 in the **Online Supplementary Document****)**. The comparison results of the Friedman test indicated that the difference was statistically significant in the error distribution of different ϵ (Friedman’s test: χ^2^ = 18.5; *P* < 0.001).

**Figure 3:**
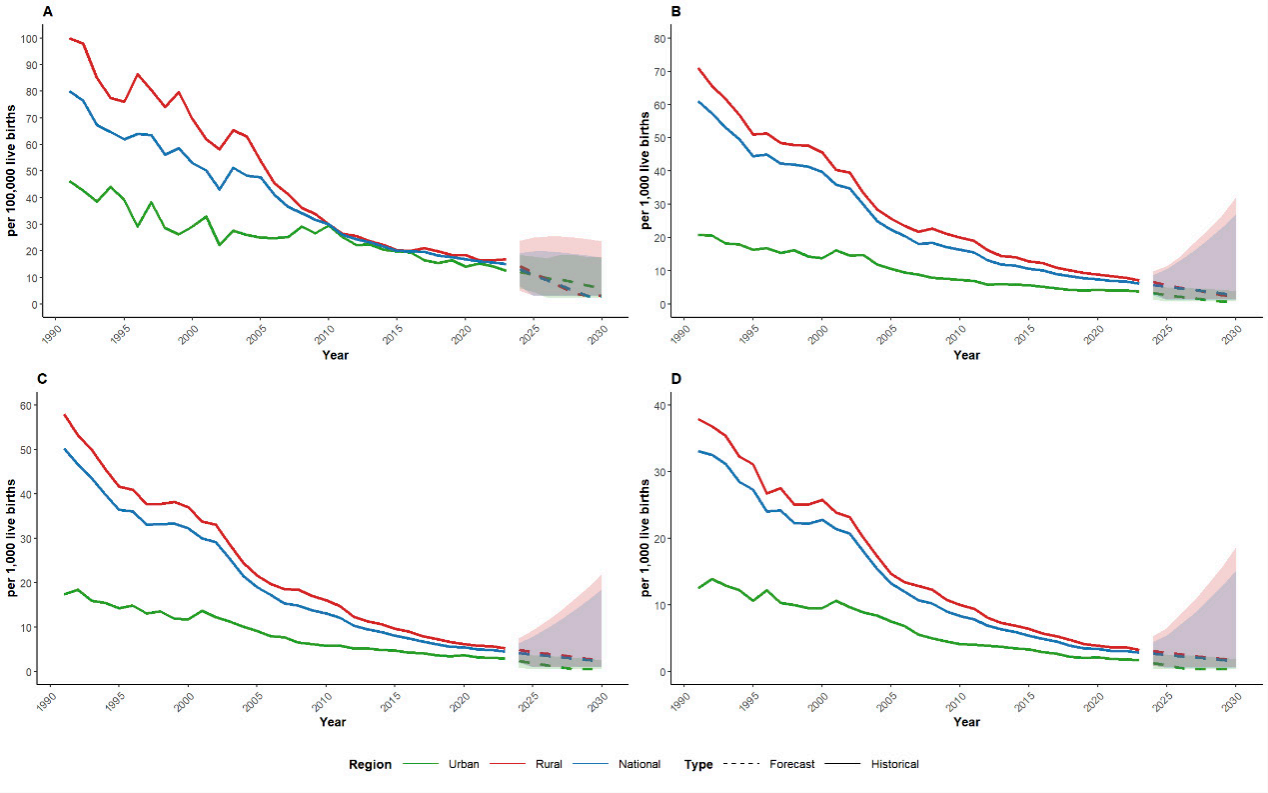
Predictive trends of health indicators at national, urban and rural levels from 2024 to 2030. **Panel A.** Predictive trends of maternal mortality ratio (MMR, per 100 000 live births). **Panel B.** Predictive trends of under-five mortality rate (U5MR, ‰). **Panel C.** Predictive trends of infant mortality rate (IMR, ‰). **Panel D.** Predictive trends of neonatal mortality rate (NMR, ‰).

We summarised predictive numbers for MMR, U5MR, IMR, and NMR from 2024 to 2030, as derived from the best-fitted ARIMA model. Forecast data indicated that maternal and child mortality rates would decline during this period, regardless of the urban, rural or nationwide setting (**Figure 3**). We estimated that the maternal and child mortality rates would approach very low, but non-zero levels by 2030, with the national MMR decreasing to 3.02 per 100 000 live births, the U5MR to 2.02‰, the IMR to 1.04‰, and the NMR to 0.56‰ (**Table 3**). Starting in 2026, we projected a potential convergence and eventual reversal of the rural-urban MMR gap. Moreover, the RDs of U5MRs, IMRs, and NMRs showed overall downward trends between 2024 and 2030. Additionally, using the GAP analysis, we found that during the forecast period, urban areas exhibited slower reductions in MMRs and NMRs compared to their rural counterparts, while demonstrating declines in U5MRs and IMRs that outpaced rural areas, with statistically significant differences observed for MMRs, NMRs, and IMRs (*P* < 0.05) but not for U5MRs (*P* > 0.05) (Table S5 in the **Online Supplementary Document**).

**Table 3:** Projection of health indicators in China from 2024 to 2030*

Indicators	Region	2024	2025	2026	2027	2028	2029	2030	Urban-rural disparities by 2030	The projected overall trend by 2030
**MMR**	National	13.07 (6.81, 19.33)	11.04 (3.02, 19.89)	9.02 (3.02, 19.86)	6.99 (3.02, 19.50)	4.96 (3.02, 18.95)	3.02 (3.02, 18.26)	3.02 (3.02, 17.46)	Narrowing	Decline
	Urban	12.20 (5.59, 18.82)	11.14 (4.32, 17.96)	9.90 (2.77, 17.03)	9.17 (2.50, 17.46)	8.15 (2.50, 16.78)	7.09 (2.50, 16.14)	6.20 (2.50, 15.87)		
	Rural	14.41 (5.02, 23.79)	11.81 (3.30, 25.09)	9.22 (3.30, 25.48)	6.62 (3.30, 25.40)	4.03 (3.30, 25.02)	3.30 (3.30, 24.43)	3.30 (3.30, 23.68)		
**U5MR**	National	5.65 (3.41, 7.89)	4.89 (1.24, 8.87)	4.25 (1.24, 10.70)	3.54 (1.24, 12.86)	3.04 (1.24, 15.22)	2.45 (1.24, 17.05)	2.02 (1.24, 19.36)	Narrowing	Decline
	Urban	3.29 (1.42, 5.16)	2.78 (0.78, 5.06)	2.24 (0.78, 4.96)	1.70 (0.78, 4.78)	1.17 (0.78, 4.56)	0.78 (0.78, 4.32)	0.78 (0.78, 4.05)		
	Rural	6.52 (4.15, 8.89)	5.48 (1.44, 9.71)	4.54 (1.44, 11.44)	3.71 (1.44, 13.78)	2.95 (1.44, 16.01)	2.20 (1.44, 17.80)	1.72 (1.44, 20.12)		
**IMR**	National	4.06 (2.31, 5.80)	3.51 (0.90, 6.80)	3.03 (0.90, 8.37)	2.36 (0.90, 10.23)	1.94 (0.90, 12.46)	1.46 (0.90, 14.32)	1.04 (0.90, 16.48)	Narrowing	Decline
	Urban	2.33 (0.80, 3.86)	1.90 (0.58, 3.68)	1.42 (0.58, 3.55)	0.96 (0.58, 3.34)	0.58 (0.58, 3.11)	0.58 (0.58, 2.86)	0.58 (0.58, 2.60)		
	Rural	4.84 (2.65, 7.04)	4.31 (1.04, 8.39)	3.72 (1.04, 10.24)	3.08 (1.04, 12.59)	2.64 (1.04, 15.12)	2.07 (1.04, 17.16)	1.65 (1.04, 19.62)		
**NMR**	National	2.48 (1.13, 3.83)	2.09 (0.56, 4.23)	1.55 (0.56, 5.07)	1.19 (0.56, 5.89)	0.75 (0.56, 6.44)	0.56 (0.56, 7.15)	0.56 (0.56, 7.62)	Narrowing	Decline
	Urban	1.25 (0.34, 2.60)	0.93 (0.34, 2.53)	0.57 (0.34, 2.47)	0.34 (0.34, 2.35)	0.34 (0.34, 2.22)	0.34 (0.34, 2.06)	0.34 (0.34, 1.89)		
	Rural	2.91 (0.97, 4.84)	2.35 (0.64, 5.19)	1.71 (0.64, 6.29)	1.07 (0.64, 7.09)	0.64 (0.64, 7.78)	0.64 (0.64, 8.43)	0.64 (0.64, 9.05)		

IMR – infant mortality rate, MMR – maternal mortality ratio, NMR – neonatal mortality rate, U5MR – under-five mortality rate

*Values presented as % (95% CI).

## DISCUSSION

Here, we investigated the trends and disparities in maternal and child mortality in China from 1991 to 2023, with a specific focus on urban-rural differences. We found significant nationwide declines in MMR, U5MR, IMR and NMR, with China surpassing relevant United Nations SDGs ahead of schedule [21].

Health equity theory aims to eliminate avoidable health disparities by ensuring equitable healthcare access [22]. Urban-rural MCH disparities arise from unequal resource distribution, economic inequality, and policy gaps within this framework. While we found notable reductions in MMRs, U5MRs, IMRs, and NMRs from 1991 to 2023, rural areas still face barriers to quality healthcare due to unequal healthcare resources and economic development [23,24]. The reductions in RD values from 1991 to 2023 reflect a significant reduction in the absolute urban-rural gap in maternal and child mortality. Nevertheless, RR values remained above 1.0 in 2023, indicating that a relative disparity persists, with mortality rates still higher in rural than in urban areas. While absolute gaps have substantially narrowed, relative disparities require continued policy attention to achieve health equity.

Although our findings for AGRs of MMRs, U5MRs, IMRs, and NMRs in urban and rural areas, as well as GAP-1 and GAP-2 values indicate that urban-rural health disparities have narrowed, key health indicators are improving more rapidly in rural regions, underscoring the need for targeted interventions in urban settings to accelerate progress toward equitable health outcomes. Compared to the AGR, the EAPC offers more detailed annual trends, particularly highlighting inter-annual fluctuations. The values of EAPCs for MMRs, U5MRs, IMRs, and NMRs observed here were significantly lower than in rural areas, especially for MMR and IMR.

We employed the ARIMA model to project health indicators up to 2030. To address the issue of potential negative values predicted by ARIMA models in scenarios of extremely low mortality, we also employed lag-transformation, rigorous model training, post-prediction value correction, and sensitivity analysis to ensure forecast validity [19]. Our findings indicated that, except for the national NMR, a smoothing parameter of ε = 0.01 ranked first in the validation set MAE for MMR, U5MR, IMR, and NMR, demonstrating its superior robustness and prediction accuracy for extremely low mortality. Projections further indicated continued nationwide improvement in health indicators. Although higher rural mortality rates, the steeper decline in rural areas was narrowing the urban-rural gap, signifying significant progress in reducing urban-rural health disparities.

China’s success in reducing maternal and child mortality may be attributed to the integration of policy intervention, technological advancement, and cultural adaptation. The implementation of the Five Strategies for Maternal and Newborn Safety (FSMNS) has enhanced maternal and infant safety by prioritising high-quality medical resources for riskier pregnancies [25]. Key initiatives, including the Reducing Maternal Mortality and Eliminating Neonatal Tetanus project [26] and the Healthy China 2030 plan, have strengthened healthcare access in underserved regions through subsidies, training, and targeted interventions. Digital platforms, such as the National Maternal and Child Health Statistical Survey System [21], have enabled precise resource allocation, directly contributing to accelerated declines in maternal-child mortality. China’s New Rural Cooperative Medical System has alleviated healthcare financial burdens by 80% through financial support and doubled institutional delivery rates in rural areas over two decades, contributing to the reduction of the urban-rural MMR ratio from 2.16 in 1991 to 1.36 in 2023 [27]. Between 1991 and 2023, a 15-fold higher rural income and a 3.2-fold increase in primary medical staffing have accelerated infant and neonatal mortality reductions [28,29]. Meanwhile, culturally responsive healthcare integration, exemplified by the Spring Bud Plan, proved pivotal for improving maternal outcomes, especially in rural populations. Using the ARIMA model, we projected a potential convergence and eventual reversal of the rural-urban MMR gap by 2026, which may be closely aligned with the county-level medical communities policy objective under China’s 14th Five-Year Plan, underscoring the cumulative impact of policy interventions and inherent time-lag effects [30]. Therefore, strengthening healthcare systems, ensuring maternal-child health service continuity during public health emergencies, and sustaining progress towards SDG targets remain policy priorities. Furthermore, while urban areas continue to deliver high-quality maternal care, their ability to achieve further substantial reductions in MMR may be constrained by the ceiling effect and the concentration of complex medical conditions. Conversely, rural regions, benefiting from large-scale policy investments, improved connectivity through telemedicine, and more complete vital registration, are experiencing faster declines [31,32]. Nevertheless, it is crucial to note that lower mortality does not equate to equivalent quality of care, and structural inequities persist, and continued efforts need to focus on strengthening frontline providers, ensuring equitable financing, and safeguarding against adverse selection.

International experiences underscore the importance of localised governance strategies in narrowing health disparities. From 1991 to 2023, India has reduced its rural IMR from 70‰ to 30‰ through the National Health Mission [33] and the Janani Suraksha Yojana [34]. However, market-driven resource allocation concurrently led to a 1.8-fold expansion of the urban-rural NMR gap, highlighting the limitations of these mechanisms. Brazil’s decentralised Unified Health System reduced urban IMR to 8‰, yet the child mortality within the Amazon area was triple the national average, a consequence of regional isolation and inadequate service accessibility [35]. Rwanda’s implementation of universal health coverage and gender equality policies significantly reduced the MMR from 1050 to 248 per 100 000 live births between 2000 and 2023 [36]. Conversely, Ethiopia’s health reforms post-peace agreement has been hindered by conflicts and drought, resulting in a high MMR of 195 per 100 000 in 2023 [37,38]. Despite Niger and Chad having improved facility deliveries via rural clinic expansion (1990s–2000s), Niger’s post-coup aid disruption (2023) sustained rural MMR above 300 per 100 000 live births [38,39], and Chad’s flood-devastated health systems (2022) reported MMR exceeding 500 per 100 000 live births [38,40]. These examples highlight the challenges in achieving the 2030 SDGs; none of these countries can meet the SDGs under current trends.

While China’s experience offers valuable insights for LMICs seeking to address urban-rural health disparities, its replicability hinges on three systemic thresholds: state capacity for resource redistribution, fiscal sustainability of grassroots healthcare networks, and the cultural adaptability of governance models [41,42]. China’s implementation of ‛tiered diagnosis and treatment’ systems and county-level medical networks has successfully decentralised high-quality healthcare resources to rural areas, significantly improving access [43]. Conversely, India’s underdeveloped regions that face structural barriers to achieving health targets, such as Uttar Pradesh, could benefit from replicating China’s approach to enhancing rural healthcare access and reducing regional disparities [44]. China’s health poverty alleviation policy has reduced the financial burden of medical expenses [45], while its village-to-village roads initiative has demonstrated the critical impact of infrastructure investments on reducing emergency response times. Brazil could replicate this model in the Amazon to enhance maternal healthcare access in remote areas by directing healthcare resources towards rural regions, strengthening digital collaboration of county-level healthcare networks (e.g. telemedicine platforms), and establishing dynamic population migration monitoring systems [46,47]. Rwanda can draw from China’s Luban Workshop to cultivate local medical talent [48], while Ethiopia needs international support to address gender inequality and medical resource scarcity [49]. China’s Belt and Road medical aid model offers a potential solution for conflict zones by providing mobile hospital networks [50]. China’s New Rural Cooperative Medical Scheme and the Rural Doctor Contract System may offer valuable insights for Niger and Chad [51–53]. These experiences highlight scalable solutions that could help LMICs close urban-rural health gaps and improve maternal and child health outcomes.

While China has achieved impressive reductions in maternal and child mortality, surpassing SDG 3 targets ahead of schedule, further progress can be informed by benchmarking against leading high-income countries. In 2023, Japan reported an MMR of 3.1 per 100 000 live births and an NMR of 0.8‰, markedly lower than China’s national MMR of 15.1 per 100 000 and NMR of 2.8‰ [38]. Notably, the USA, despite its high healthcare expenditure, recorded an MMR of 16.6 per 100 000, a figure comparable to China’s national average [38]. However, this aggregate rate conceals racial disparities, as the MMR among black women exceeds 40 per 100 000, nearly three times that of white women. This disparity underscores that low national mortality rates do not inherently reflect equitable healthcare outcomes [54]. China now faces a similar inflexion point in which, although its overall rates have improved significantly, substantial urban–rural gaps persist, particularly in NMR and IMR. To advance toward the standards exemplified by Japan, future efforts should shift from broad coverage to precision quality improvement, ensuring skilled birth attendance, emergency obstetric care access, and integrated perinatal mental health services reach every woman, especially in rural and marginalised communities. Additionally, strengthening primary care integration, leveraging digital health technologies for remote monitoring, and addressing social determinants such as maternal age, nutrition, and education will be critical in closing the final gap [55].

### Limitations

Although the NBS retrospectively adjusts for definitional changes, we could not account for unmeasured confounders such as shifts in surveillance sensitivity or urban-rural reclassification, which may have affected trend interpretation. Researchers should consider these factors when generalising findings to other settings with less centralised data revision mechanisms. Furthermore, our projections statistically extrapolate current trends; any apparent reversals in urban-rural gaps should be interpreted as hypothetical scenarios, rather than established trends.

## CONCLUSIONS

China has made significant progress in MCH from 1991 to 2023, with mortality rates falling steadily in both urban and rural areas. While absolute gaps have substantially narrowed, relative disparities require continued policy attention to achieve health equity. Future MCH services should shift from broad coverage to precision quality improvement. These experiences also provide important lessons for LMICs undergoing rapid urbanization, underscoring the necessity of synchronizing the development of urban and rural healthcare system to promote equitable and accessible health outcomes for all.

## Additional material


Online Supplementary Document
