# Supplementary material for: Reducing urban-rural disparities in maternal and child mortality in China: a 33-year analysis and projection to 2030
Source: J Glob Health. 2026 Jan 12;16:04011. doi: 10.7189/jogh.16.04011 (PMC12796867; doi:10.7189/jogh.16.04011)
Supplement: Online Supplementary Document [file jogh-16-04011-s001.pdf]

Supplement to: Rao Z, Liu B, Li D, Qing Z, Lu Y, Yin D, Li S, Cheng K, Xiao Y, Dai Q. Reducing urban-rural disparities in maternal and child mortality in China: a 33-year analysis and projection to 2030. J Glob Health. 2026;16:04011.

Included files

TABLES

Table S1. The data of health indicators in China from 1991 to 2023.

Table S2. The RD and RR of health indicators in China from 1991 to 2023.

Table S3. Comparison of actual and predictive values for MMR, U5MR, IMR, NMR in 2023.

Table S4. Prediction errors of each health indicator under different ϵ values.

Table S5. The urban-rural disparities of health indicators from 2024 to 2030.

FIGURES

Figure S1. The results of residual analysis for MMR at the national, urban, and rural levels.

Figure S2. The results of residual analysis for U5MR at the national, urban, and rural levels.

Figure S3. The results of residual analysis for IMR at the national, urban, and rural levels.

Figure S4. The results of residual analysis for NMR at the national, urban, and rural levels.

Table S1. The data of health indicators in China from 1991 to 2023

Year	MMR (per 100,000)			U5MR (‰)			IMR (‰)			NMR (‰)		
	National	Urban	Rural	National	Urban	Rural	National	Urban	Rural	National	Urban	Rural
**1991**	80.00	46.30	100.00	61.00	20.90	71.10	50.20	17.30	58.00	33.10	12.50	37.90
**1992**	76.50	42.70	97.90	57.40	20.70	65.60	46.70	18.40	53.20	32.50	13.90	36.80
**1993**	67.30	38.50	85.10	53.10	18.30	61.60	43.60	15.90	50.00	31.20	12.90	35.40
**1994**	64.80	44.10	77.50	49.60	18.00	56.90	39.90	15.50	45.60	28.50	12.20	32.30
**1995**	61.90	39.20	76.00	44.50	16.40	51.10	36.40	14.20	41.60	27.30	10.60	31.10
**1996**	63.90	29.20	86.40	45.00	16.90	51.40	36.00	14.80	40.90	24.00	12.20	26.70
**1997**	63.60	38.30	80.40	42.30	15.50	48.50	33.10	13.10	37.70	24.20	10.30	27.50
**1998**	56.20	28.60	74.10	42.00	16.20	47.90	33.20	13.50	37.70	22.30	10.00	25.10
**1999**	58.70	26.20	79.70	41.40	14.30	47.70	33.30	11.90	38.20	22.20	9.50	25.10
**2000**	53.00	29.30	69.60	39.70	13.80	45.70	32.20	11.80	37.00	22.80	9.50	25.80
**2001**	50.20	33.10	61.90	35.90	16.30	40.40	30.00	13.60	33.80	21.40	10.60	23.90
**2002**	43.20	22.30	58.20	34.90	14.60	39.60	29.20	12.20	33.10	20.70	9.70	23.20
**2003**	51.30	27.60	65.40	29.90	14.80	33.40	25.50	11.30	28.70	18.00	8.90	20.10
**2004**	48.30	26.10	63.00	25.00	12.00	28.50	21.50	10.10	24.50	15.40	8.40	17.30
**2005**	47.70	25.00	53.80	22.50	10.70	25.70	19.00	9.10	21.60	13.20	7.50	14.70
**2006**	41.10	24.80	45.50	20.60	9.60	23.60	17.20	8.00	19.70	12.00	6.80	13.40
**2007**	36.60	25.20	41.30	18.10	9.00	21.80	15.30	7.70	18.60	10.70	5.50	12.80
**2008**	34.20	29.20	36.10	18.50	7.90	22.70	14.90	6.50	18.40	10.20	5.00	12.30
**2009**	31.90	26.60	34.00	17.20	7.60	21.10	13.80	6.20	17.00	9.00	4.50	10.80
**2010**	30.00	29.70	30.10	16.40	7.30	20.10	13.10	5.80	16.10	8.30	4.10	10.00
**2011**	26.10	25.20	26.50	15.60	7.10	19.10	12.10	5.80	14.70	7.80	4.00	9.40
**2012**	24.50	22.20	25.60	13.20	5.90	16.20	10.30	5.20	12.40	6.90	3.90	8.10
**2013**	23.20	22.40	23.60	12.00	6.00	14.50	9.50	5.20	11.30	6.30	3.70	7.30
**2014**	21.70	20.50	22.20	11.70	5.90	14.20	8.90	4.80	10.70	5.90	3.50	6.90
**2015**	20.10	19.80	20.20	10.70	5.80	12.90	8.10	4.70	9.60	5.40	3.30	6.40
**2016**	19.90	19.50	20.00	10.20	5.20	12.40	7.50	4.20	9.00	4.90	2.90	5.70
**2017**	19.60	16.60	21.10	9.05	4.84	10.94	6.77	4.15	7.94	4.48	2.65	5.30
**2018**	18.30	15.50	19.90	8.40	4.40	10.20	6.10	3.60	7.30	3.90	2.20	4.70
**2019**	17.80	16.50	18.60	7.80	4.10	9.40	5.60	3.40	6.60	3.50	2.00	4.10
**2020**	16.90	14.10	18.50	7.50	4.40	8.90	5.40	3.60	6.20	3.40	2.10	3.90
**2021**	16.10	15.40	16.50	7.10	4.10	8.50	5.00	3.20	5.80	3.10	1.90	3.60
**2022**	15.70	14.30	16.60	6.80	4.20	8.00	4.90	3.10	5.70	3.10	1.80	3.60
**2023**	15.10	12.50	17.00	6.20	3.90	7.20	4.50	2.90	5.20	2.80	1.70	3.20

Abbreviations: MMR: maternal mortality ratio; U5MR: under-five mortality rate; IMR: infant mortality rate; NMR: neonatal mortality rate.

Table S2. The RD and RR of health indicators in China from 1991 to 2023

Year	MMR		U5MR		IMR		NMR	
	RD (per 100,000)	RR	RD (‰)	RR	RD (‰)	RR	RD (‰)	RR
**1991**	53.70	2.16	50.20	3.40	40.70	3.35	25.40	3.03
**1992**	55.20	2.29	44.90	3.17	34.80	2.89	22.90	2.65
**1993**	46.60	2.21	43.30	3.37	34.10	3.14	22.50	2.74
**1994**	33.40	1.76	38.90	3.16	30.10	2.94	20.10	2.65
**1995**	36.80	1.94	34.70	3.12	27.40	2.93	20.50	2.93
**1996**	57.20	2.96	34.50	3.04	26.10	2.76	14.50	2.19
**1997**	42.10	2.10	33.00	3.13	24.60	2.88	17.20	2.67
**1998**	45.50	2.59	31.70	2.96	24.20	2.79	15.10	2.51
**1999**	53.50	3.04	33.40	3.34	26.30	3.21	15.60	2.64
**2000**	40.30	2.38	31.90	3.31	25.20	3.14	16.30	2.72
**2001**	28.80	1.87	24.10	2.48	20.20	2.49	13.30	2.25
**2002**	35.90	2.61	25.00	2.71	20.90	2.71	13.50	2.39
**2003**	37.80	2.37	18.60	2.26	17.40	2.54	11.20	2.26
**2004**	36.90	2.41	16.50	2.38	14.40	2.43	8.90	2.06
**2005**	28.80	2.15	15.00	2.40	12.50	2.37	7.20	1.96
**2006**	20.70	1.83	14.00	2.46	11.70	2.46	6.60	1.97
**2007**	16.10	1.64	12.80	2.42	10.90	2.42	7.30	2.33
**2008**	6.90	1.24	14.80	2.87	11.90	2.83	7.30	2.46
**2009**	7.40	1.28	13.50	2.78	10.80	2.74	6.30	2.40
**2010**	0.40	1.01	12.80	2.75	10.30	2.78	5.90	2.44
**2011**	1.30	1.05	12.00	2.69	8.90	2.53	5.40	2.35
**2012**	3.40	1.15	10.30	2.75	7.20	2.38	4.20	2.08
**2013**	1.20	1.05	8.50	2.42	6.10	2.17	3.60	1.97
**2014**	1.70	1.08	8.30	2.41	5.90	2.23	3.40	1.97
**2015**	0.40	1.02	7.10	2.22	4.90	2.04	3.10	1.94
**2016**	0.50	1.03	7.20	2.38	4.80	2.14	2.80	1.97
**2017**	4.50	1.27	6.10	2.26	3.79	1.91	2.65	2.00
**2018**	4.40	1.28	5.80	2.32	3.70	2.03	2.50	2.14
**2019**	2.10	1.13	5.30	2.29	3.20	1.94	2.10	2.05
**2020**	4.40	1.31	4.50	2.02	2.60	1.72	1.80	1.86
**2021**	1.10	1.07	4.40	2.07	2.60	1.81	1.70	1.89
**2022**	2.30	1.16	3.80	1.90	2.60	1.84	1.80	2.00
**2023**	4.50	1.36	3.30	1.85	2.30	1.79	1.50	1.88

Abbreviations: MMR: maternal mortality ratio; U5MR: under-five mortality rate; IMR: infant mortality rate; NMR: neonatal mortality rate; RD: rate difference; RR: rate ratio.

Table S3. Comparison of actual and predictive values for MMR, U5MR, IMR, NMR in 2023

Indicators	Region	Actual	Predicted	95% CI	Difference (Actual-Predicted)
**MMR (per 100,000)**	**National**	15.10	13.63	(7.28, 19.97)	1.47
	**Urban**	12.50	12.91	(6.49, 19.33)	-0.41
	**Rural**	17.00	13.91	(4.43, 23.39)	3.09
**U5MR (‰)**	**National**	6.20	6.44	(3.40, 9.49)	-0.24
	**Urban**	3.90	3.45	(1.56, 5.35)	0.45
	**Rural**	7.20	7.73	(4.50, 10.97)	-0.53
**IMR (‰)**	**National**	4.50	4.67	(2.45, 6.88)	-0.17
	**Urban**	2.90	2.47	(0.92, 4.02)	0.43
	**Rural**	5.20	5.42	(2.72, 8.12)	-0.22
**NMR (‰)**	**National**	2.80	2.91	(1.14, 4.68)	-0.11
	**Urban**	1.70	1.34	(0.36, 2.71)	0.36
	**Rural**	3.20	3.39	(1.11, 5.68)	-0.19

Abbreviations: MMR: maternal mortality ratio; U5MR: under-five mortality rate; IMR: infant mortality rate; NMR: neonatal mortality rate; 95% CI: 95% confidence interval.

Table S4. Prediction errors of each health indicator under different ϵ values

Indicators	ϵ	MAE (Training)	RMSE (Training)	MAE (Validation)	RMSE (Validation)	Optimal ranking*
**National MMR**	0.01	2.070	3.158	0.741	0.795	1
	0.10	2.069	3.158	0.748	0.803	2
	1.00	2.066	3.157	0.817	0.878	3
**Urban MMR**	0.01	2.754	3.594	0.754	0.857	1
	0.10	2.754	3.595	0.754	0.855	2
	1.00	2.754	3.596	0.757	0.841	3
**Rural MMR**	0.01	3.197	4.785	0.860	1.154	1
	0.10	3.197	4.785	0.863	1.161	2
	1.00	3.202	4.783	0.900	1.236	3
**National U5MR**	0.01	1.031	1.412	0.308	0.355	1
	0.10	1.030	1.411	0.317	0.367	2
	1.00	1.021	1.401	0.420	0.478	3
**Urban U5MR**	0.01	0.709	0.991	0.449	0.502	1
	0.10	0.709	0.991	0.456	0.510	2
	1.00	0.710	0.989	0.516	0.585	3
**Rural U5MR**	0.01	1.227	1.655	0.175	0.211	1
	0.10	1.227	1.655	0.184	0.220	2
	1.00	1.227	1.649	0.271	0.316	3
**National IMR**	0.01	0.738	1.089	0.242	0.282	1
	0.10	0.737	1.087	0.252	0.294	2
	1.00	0.725	1.070	0.349	0.413	3
**Urban IMR**	0.01	0.489	0.765	0.176	0.195	1
	0.10	0.489	0.764	0.183	0.201	2
	1.00	0.496	0.758	0.246	0.267	3
**Rural IMR**	0.01	0.907	1.267	0.157	0.196	1
	0.10	0.907	1.266	0.163	0.204	2
	1.00	0.910	1.254	0.237	0.302	3
**National NMR**	0.01	0.614	0.881	0.404	0.474	3
	0.10	0.666	0.941	0.138	0.163	1
	1.00	0.652	0.916	0.234	0.273	2
**Urban NMR**	0.01	0.458	0.769	0.105	0.110	1
	0.10	0.458	0.767	0.112	0.118	2
	1.00	0.457	0.759	0.179	0.194	3
**Rural NMR**	0.01	0.815	1.142	0.132	0.153	1
	0.10	0.814	1.139	0.135	0.152	2
	1.00	0.808	1.121	0.155	0.192	3

Abbreviations: MMR: maternal mortality ratio; U5MR: under-five mortality rate; IMR: infant mortality rate; NMR: neonatal mortality rate; MAE: mean absolute error; RMSE: root mean squared error.

Table S5. The urban-rural disparities of health indicators from 2024 to 2030

Indicators		2024	2025	2026	2027	2028	2029	2030	Urban AGR	Rural AGR	GAP-1 (95% CI)	GAP-2 (95% CI)	Z_{GAP-1}	Z_{GAP-2}	P_{GAP-1}	P_{GAP-2}
**MMR**	**RD (per 100,000)**	2.21	0.67	-0.68	-2.55	-4.12	-3.79	-2.90	-10.67%	-21.78%	0.49 (0.44, 0.54)	-51.01% (-60.80%, 41.20%)	-14.26	-10.20	< 0.001	< 0.001
	**RR**	1.18	1.06	0.93	0.72	0.49	0.47	0.53								
**U5MR**	**RD (‰)**	3.23	2.70	2.30	2.01	1.78	1.42	0.94	-21.33%	-19.92%	1.07 (0.97, 1.18)	7.08% (-2.80%, 16.80%)	1.35	1.40	0.176	0.162
	**RR**	1.98	1.97	2.03	2.18	2.52	2.82	2.21								
**IMR**	**RD (‰)**	2.51	2.41	2.30	2.12	2.06	1.49	1.07	-20.89%	-16.42%	1.27 (1.15, 1.40)	27.22% (17.20%, 36.80%)	4.78	5.40	< 0.001	< 0.001
	**RR**	2.08	2.27	2.62	3.21	4.55	3.57	2.84								
**NMR**	**RD (‰)**	1.66	1.42	1.14	0.73	0.30	0.30	0.30	-19.51%	-22.31%	0.87 (0.79, 0.96)	-12.55% (-22.30%, -2.70%)	-2.78	-2.50	0.005	0.012
	**RR**	2.33	2.53	3.00	3.15	1.88	1.88	1.88								

Note: GAP-1 reflects the multiple relationship of urban and rural growth rates, making it appropriate for long-term trend analysis. GAP-2 highlights the difference in improvement speed between urban and rural areas, rendering it suitable for evaluating policy effectiveness. MMR: maternal mortality ratio; U5MR: under-five mortality rate; IMR: infant mortality rate; NMR: neonatal mortality rate; RD: rate difference; RR: rate ratio; AGR: average growth rate; 95% CI: 95% confidence interval.

Figure S1. The results of residual analysis for MMR at the national, urban, and rural levels

National MMR

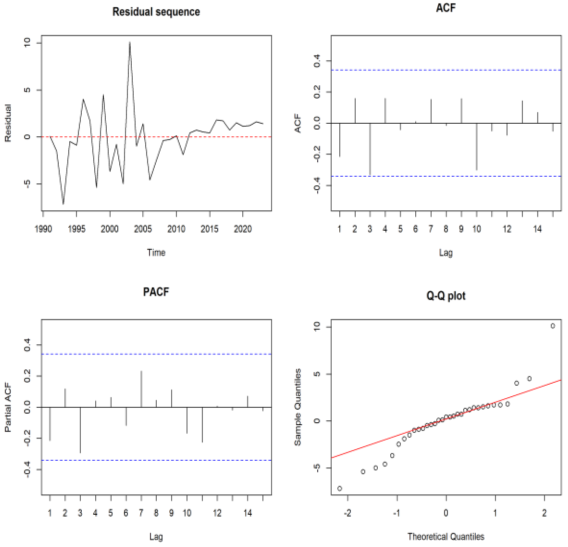

Urban MMR

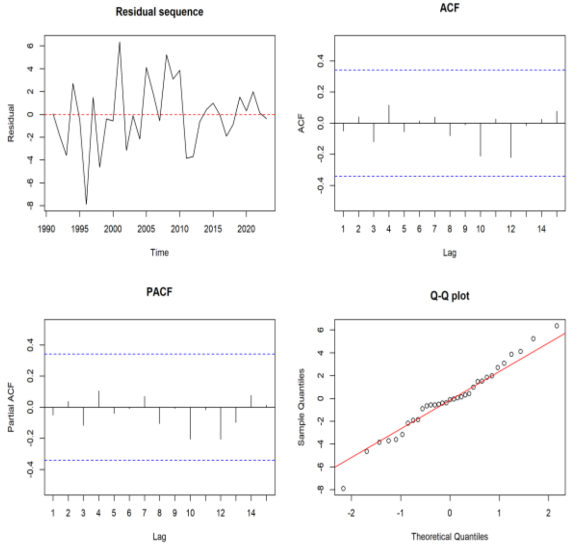

Rural MMR

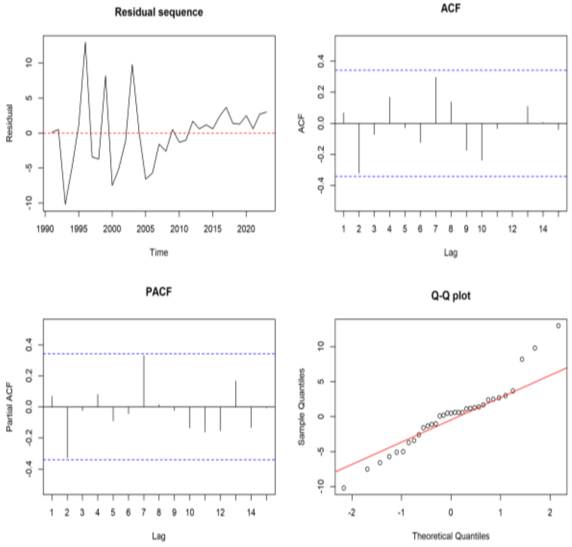

Abbreviations: MMR: maternal mortality ratio; ACF: autocorrelation function; PACF: partial autocorrelation function; Q-Q plot: quantile-quantile plot.

Figure S2. The results of residual analysis for U5MR at the national, urban, and rural levels

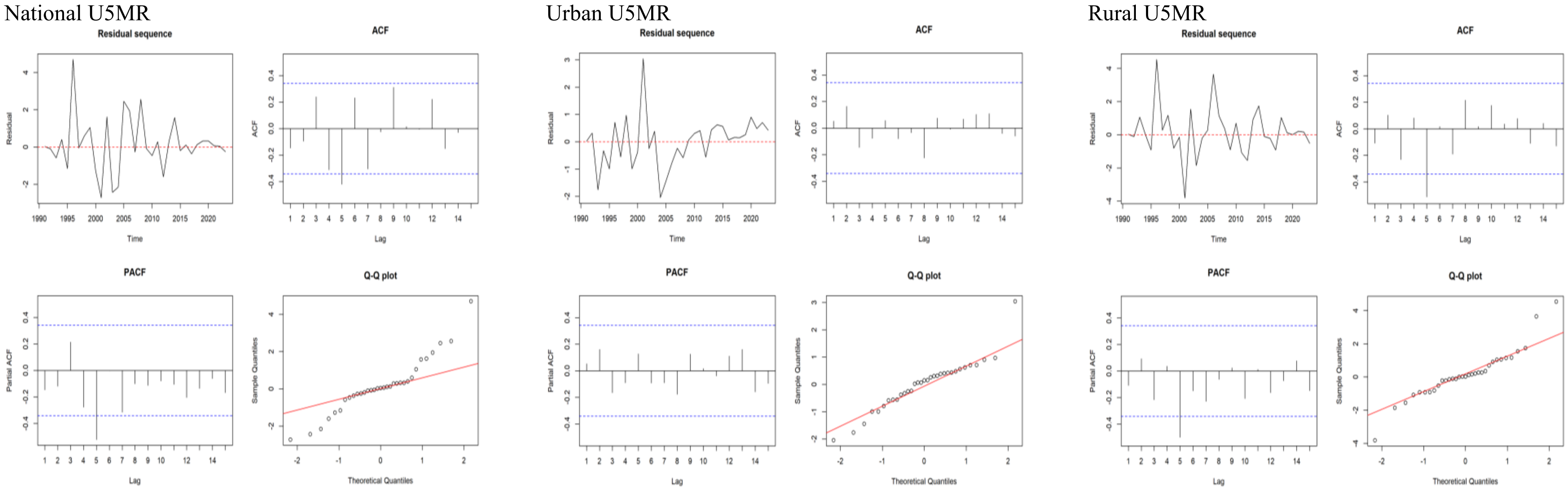

Abbreviations: U5MR: under-five mortality rate; ACF: autocorrelation function; PACF: partial autocorrelation function; Q-Q plot: quantile-quantile plot.

Figure S3. The results of residual analysis for IMR at the national, urban, and rural levels

National IMR

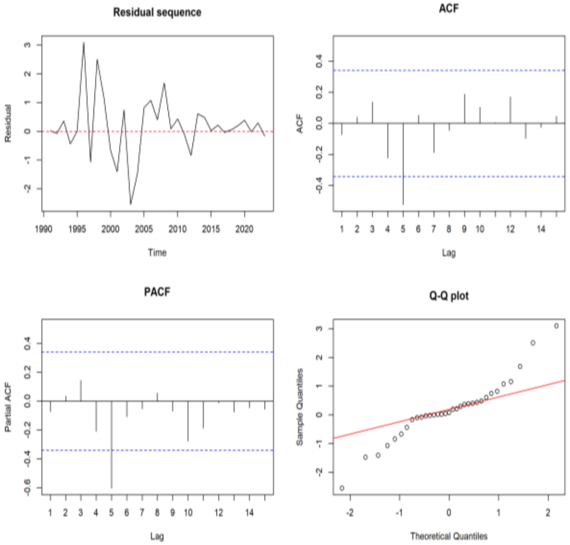

Urban IMR

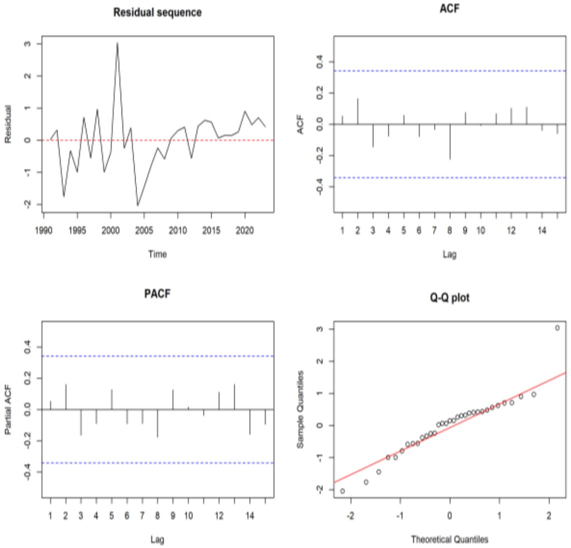

Rural IMR

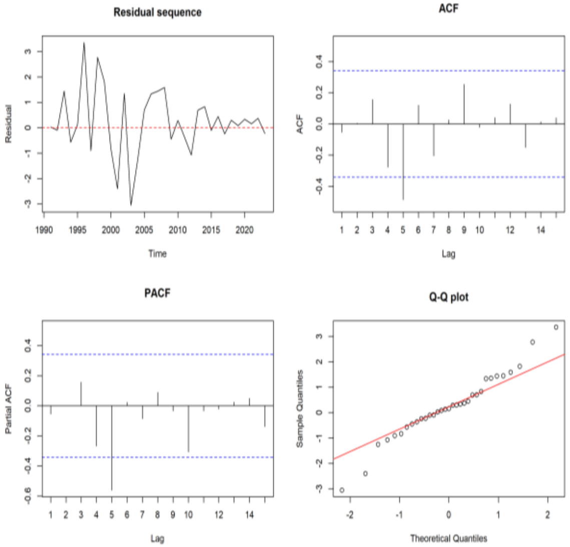

Abbreviations: IMR: infant mortality rate; ACF: autocorrelation function; PACF: partial autocorrelation function; Q-Q plot: quantile-quantile plot.

Figure S4. The results of residual analysis for NMR at the national, urban, and rural levels

National NMR | Urban NMR | Rural NMR

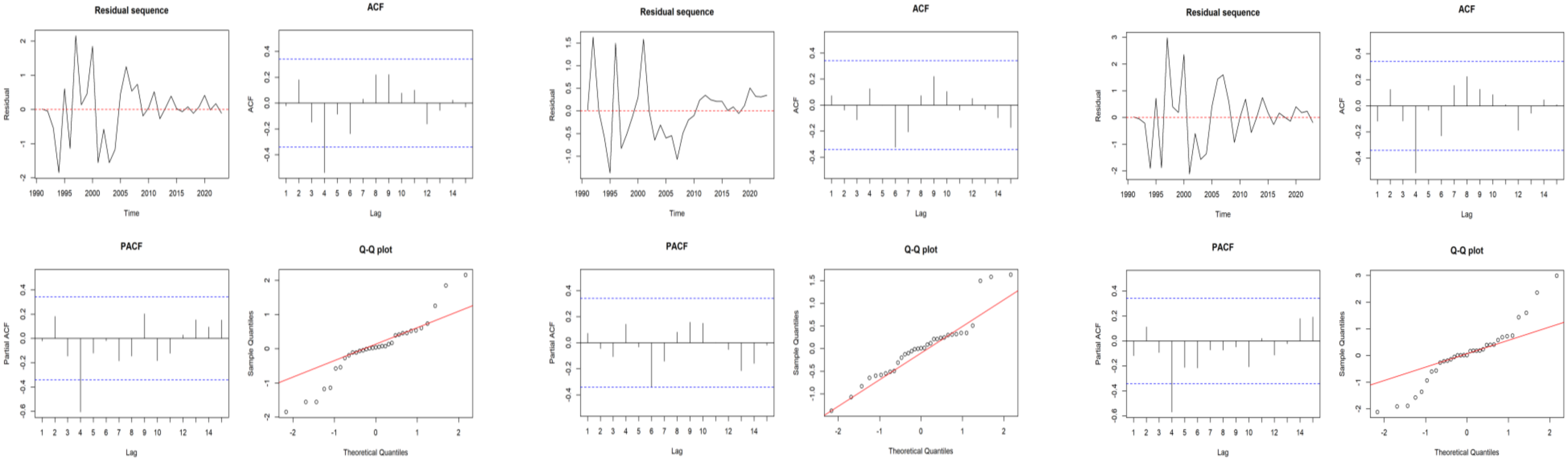

Abbreviations: NMR: neonatal mortality rate; ACF: autocorrelation function; PACF: partial autocorrelation function; Q-Q plot: quantile-quantile plot.
